# Supplementary material for: A quantitative description of light-limited cyanobacterial growth using flux balance analysis
Source: PLoS Comput Biol. 2024 Aug 5;20(8):e1012280. doi: 10.1371/journal.pcbi.1012280 (PMC11326710; doi:10.1371/journal.pcbi.1012280)
Supplement: S1 Text — Additional figures and tables as referenced in the manuscript. (PDF) [file pcbi.1012280.s001.pdf]

Supplementary Information S1 Text
"A quantitative description of light-limited cyanobacterial growth using flux balance analysis"
by Höper et al. (2024)

List of Supplemental Files

S1 Text (this file). Supplementary Text (PDF). Additional figures and tables as referenced in the manuscript.

S1 Table. The reconstructed network (XLSX). A table/text version of the reconstructed network in xlsx format.

S2 Table. Added reactions (CSV). A full list of added reactions compared to the model of Knoop et al. (2015) [4].

S1 File. The main network reconstruction (SBML). The genome-scale reconstruction of *Synechocystis* sp. PCC 6803 as an SBML file.

S2 File. Constrained network (SBML). A model of *Synechocystis* sp. PCC 6803 as an SBML file with explicit flux constraints.

S3 File. The MEMOTE report (HTML).

S4 File. Script archive (TAR.GZ). A compressed archive of scripts for simulations and to generate the figures.

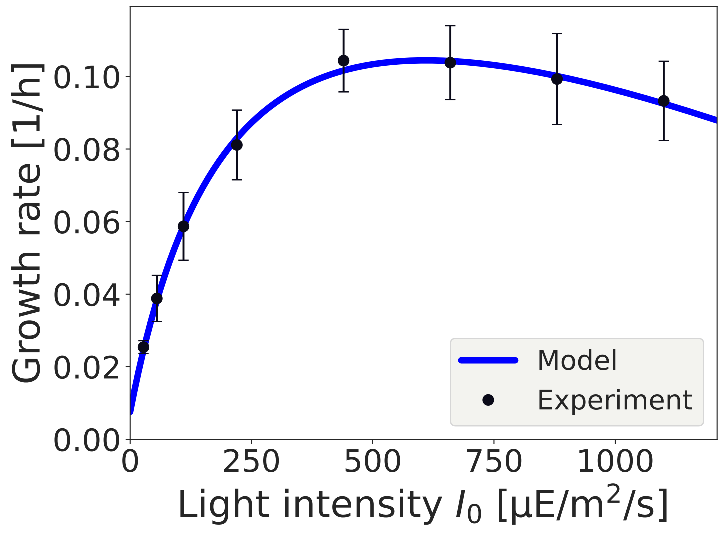

Figure A: A fit of the growth rate using the static BOF. Methods identical to Figure 3 in the main text but with the static BOF for all light intensities. The detailed composition of the BOF has no major impact on the quality of the fit of the model to the measured growth rate.

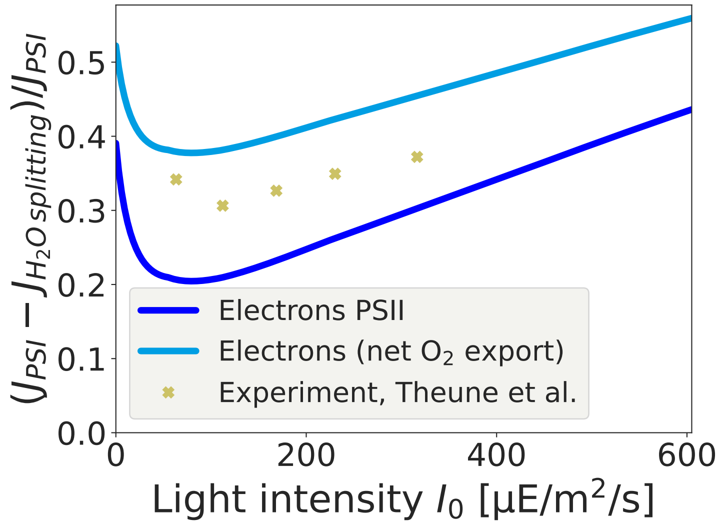

Figure B: Predicted linear (LET) versus cyclic electron transport (CET) as described in the main text. Electrons from PSII (water splitting) are counted either directly from the flux at PSII (lower line) or are estimated from net O_2 export (4 electrons per O_2, upper line). The latter (upper line) corresponds to the experimental quantification of Theune et al. [1]. We note that light intensities in both setups cannot necessarily be directly compared, since the effective light intensity also depends on culture density and vessel geometry, hence the x-axis (light intensity) may be shifted.

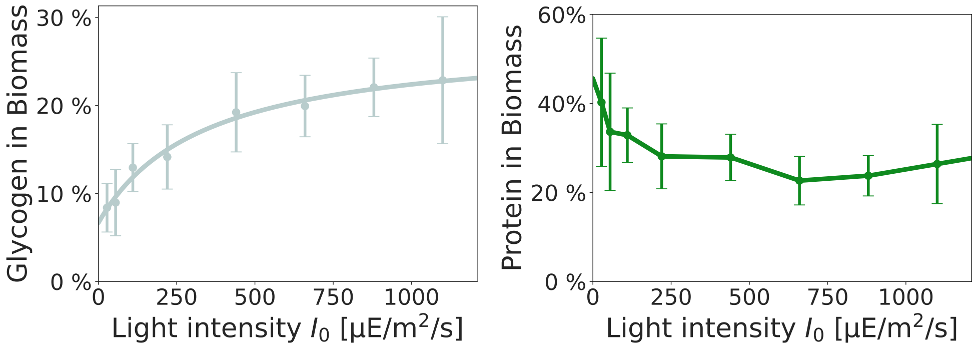

Figure C: The experimentally determined glycogen and protein mass fractions used in the light-dependent BOF. For a model evaluation across the entire range of light intensities, the glycogen mass fraction was fitted to a Monod function with offset, i.e., $glyc = a\, I_0/(b + I_0) + c$ with $a = 0.21$, $b = 336.56$, $c = 0.07$. The protein mass fraction was linearly interpolated between experimental values. All data as originally reported in [2].

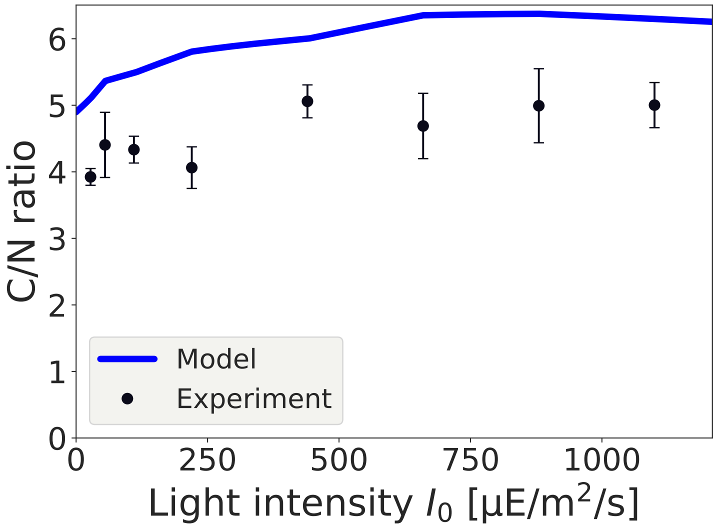

Figure D: Comparison of the C:N ratio obtained from the model versus experimental values reported by Zavřel et al. [2].

Table A: A static biomass objective function (BOF), adopted from [3, 4]. The static BOF is used as a reference. Shown are the amounts of coarse-grained components.

Stoichiometry	Metabolite	Name
-0.52	`bm_pro_c`	Protein component of BOF
-0.03	`bm_dna_c`	DNA component of BOF
-0.17	`bm_rna_c`	RNA component of BOF
-0.06	`bm_cw_c`	Cell wall component of BOF
-0.12	`bm_memlip_c`	Lipid component of BOF
-0.03	`bm_sol_c`	Soluble pool component of BOF
-0.01	`bm_ion_c`	Ion component of BOF
-0.02	`bm_pigm_c`	Pigment component of BOF
-0.03	`bm_glycogen_c`	Glycogen component of BOF
-53.35	`atp_c`	
-53.35	`h2o_c`	
1.0	`biomass_e`	Biomass (BOF)
53.35	`adp_c`	
53.35	`pi_c`	Orthophosphate
53.35	`h_c`	

Table B: The elemental composition as calculated from the total sum of fluxes of the exchange reactions of the model. The composition is estimated using the BOF for a light intensity $I_0 = 660$ µE/m^2/s corresponding to a growth rate of 0.10 h^{-1} with a mass fraction of 23% protein and 20% glycogen.

Element	Amount	Mass
H	64.272 mmol	64.8 mg
O	19.084 mmol	305.3 mg
C	40.846 mmol	490.6 mg
Na	0.005 mmol	0.1 mg
N	6.482 mmol	90.8 mg
S	0.124 mmol	4.0 mg
P	0.893 mmol	27.7 mg
Mn	0.004 mmol	0.2 mg
K	0.222 mmol	8.7 mg
Zn	0.004 mmol	0.3 mg
Co	0.004 mmol	0.3 mg
Fe	0.019 mmol	1.0 mg
Mg	0.036 mmol	0.9 mg
Ca	0.006 mmol	0.2 mg
Cu	0.004 mmol	0.3 mg
Mo	0.004 mmol	0.4 mg
**tot. mass**		**995.6 mg**

Table C: According to the stoichiometric reconstruction, (maximal) growth necessarily results in the synthesis and excretion of three byproducts. The table provides the products and their excretion flux rate (in units mmol/gCDM/h) for a reference growth rate of 1.0 h^{-1} and a reference CO_2 fixation flux of 41.4 mmol/gCDM/h. The synthesis of byproducts involves three reactions. Dialurate results from the oxidative reduction of FMN (Riboflavin-5-phosphate), necessary for vitamin B12 synthesis. Synthesis of 4-Amino-2-methyl-5-phosphomethylpyrimidine (which is required for synthesis of thiamine diphosphate) results in synthesis of CO and 5'-Deoxyadenosine. The latter is then degraded to 5-deoxy-d-ribose.

Name	MetID	ChEBI	Flux
5-Deoxy-D-ribose	`5drib_e`	62012	$2.32 \cdot 10^{-4}$
Dialurate	`dialurate_e`	76452	$2.32 \cdot 10^{-4}$
Carbon monoxide	`co_e`	17245	$2.32 \cdot 10^{-4}$

ID	Stoichiometry
FMNRx2	`fmnh2_c + nadp_c <-> fmn_c + 2.0 h_c + nadph_c`
RI0011	`fmnh2_c + o2_c --> dialurate_c + dmbzid_c + e4p_c + 2.0 h_c`
AHMMPS2	`air_c + amet_c <-> 4a2mpmpmd_c + co_c + dad_5_c + ...`
5DOAN	`dad_5_c + h2o_c --> 5drib_c + ade_c`

References

[1] Theune ML, Hildebrandt S, Steffen-Heins A, Bilger W, Gutekunst K, Appel J. In-vivo quantification of electron flow through photosystem I – Cyclic electron transport makes up about 35% in a cyanobacterium. Biochimica et Biophysica Acta (BBA) - Bioenergetics. 2021;1862(3):148353. doi:https://doi.org/10.1016/j.bbabio.2020.148353.

[2] Zavřel T, Faizi M, Loureiro C, Poschmann G, Stühler K, Sinetova M, et al. Quantitative insights into the cyanobacterial cell economy. eLife. 2019;8:e42508. doi:10.7554/eLife.42508.

[3] Knoop H, Gründel M, Zilliges Y, Lehmann R, Hoffmann S, Lockau W, et al. Flux balance analysis of cyanobacterial metabolism: the metabolic network of Synechocystis sp. PCC 6803. PLoS Comput Biol. 2013;9(6):e1003081.

[4] Knoop H, Steuer R. A Computational Analysis of Stoichiometric Constraints and Trade-Offs in Cyanobacterial Biofuel Production. Frontiers in Bioengineering and Biotechnology. 2015;3. doi:10.3389/fbioe.2015.00047.
