# Supplementary material for: A quantitative description of light-limited cyanobacterial growth using flux balance analysis
Source: PLoS Comput Biol. 2024 Aug 5;20(8):e1012280. doi: 10.1371/journal.pcbi.1012280 (PMC11326710; doi:10.1371/journal.pcbi.1012280)
Supplement: Attachment — Submitted filename: Response-Reviewers - PCOMPBIOL-D-24-00071-FINAL.pdf [file pcbi.1012280.s008.pdf]

Response to the reports of the reviewers on the manuscript "A quantitative description of light-limited cyanobacterial growth using flux balance analysis" submitted to PLoS Computational Biology (PCOMPBIOL-D-24-00071)

We would like to thank all reviewers for their careful evaluation of our manuscript. We are pleased to note that the reviewers consider the text to be relevant, well written, and of importance and high interest to the audience of PLoS CB. We have carefully revised the manuscript according to the suggestions of the reviewers. We hope that, given the requested changes and revisions, our manuscript is now suitable for publication in PLoS Computational Biology.

Below we give a detailed response to all comments and describe the respective changes in the manuscript. The original reviews are included unabridged, our response is indented and highlighted in blue. All changes are also marked in the original manuscript.

Overall changes with respect to the Supplemental Files:

- As noted by several reviewers there was a technical problem with the Jupyter notebook. The notebook was intended to automatically download the required data for parameter fitting from the eLife website. We think this may have caused problems ("ValueError: Excel file format cannot be determined").

We now instead provide two Jupyter notebooks: One that simulates the model and generates the respective figures for a given set of parameters embedded in the file (parameters as used in the manuscript). In addition, we provide an additional Jupyter notebook that carries out the parameter estimation, with the data directly embedded into the file. We note that the 2nd notebook takes more time to execute as the parameter fitting requires additional computations. We recommend using the first notebook (*plots_only.ipnb*) to explore the model.

- We carefully revised the metabolic reconstruction. In particular, the original submitted version used neutral elemental formulas for all metabolites (as previously in Knoop, 2013, 2015, and following KEGG), resulting in inconsistencies with the stated charges of metabolites (as noted by reviewer #1). The revised network resolves the issue and uses protonated states for all metabolites. In the process, we also resolved several (previously pooled) fatty acids into individual compounds, hence the network is now slightly larger (920 reactions). The changes in fatty acid metabolism have only minor impact on simulation results.

- As noted by reviewer #1, within the submitted network files, there was a problem with gene-protein-reaction (GPR) associations. This was due to an error in the script: whenever a reaction did not have an associated annotation, the script instead retained the GPR from the previous reaction, resulting in incorrect (and at times confusing) GPR. We apologize for this mistake

We note that these corrections do not affect the overall results reported in the manuscript.

All further changes are detailed below.

Comments to the Authors:

Reviewer #1:

In the manuscript "A quantitative description of light-limited cyanobacterial growth using flux balance analysis" submitted for publication in PLOS Comp Bio, the authors present a genome-scale metabolic model for the cyanobacterium Synechocystis sp. PCC 6803 to understand the effect of light intensity on the growth rate. The model includes several updates from previously reconstructed models from the same research group. To understand how light intensity (and composition) affects growth rate, the model was coupled to a comprehensive mathematical description of light utilization and photoinhibition dependent on the light intensity (and composition; red + blue light). Through experimental measurements, free parameters in this mathematical description were determined, as well parameters such as NGAM, so the uptake of light was constrained accordingly. The simulated growth rates, oxygen production, and ATP/NADPH ratios agreed with experimental values.

MAJOR COMMENTS:

As the authors established a phenomenological effect of light on the growth rate, it is expected that aspects of Synechocystis biology are disregarded from the model and from the subsequent discussion of the results. For instance, the relief of electron overflow through the terminal oxidases and the Mehler reactions was assumed constant for every light intensity. Readers would benefit from a discussion on how they could affect current simulation, and how further dissect their specific role as tolerance mechanisms to (high) light intensities, if they play any significant role and if they are adaptable through active gene expression.

> → We fully agree. We note that our approach remains within the general framework (and therefore also within the limits) of constraint-based modeling. That is, flow through the terminal oxidase and through other "overflow" reactions are provided as a constraint and do not emerge from the model itself. The respective constraints can be provided based on data, including (to some extent) expression data or, as done here, using parameter fitting. We note that FBA is limited in its capability to simulate, e.g. sudden increases in light intensities (although there might be suitable approaches available, e.g. minimal metabolic adjustment, MOMA, but such an analysis is beyond the scope of the current work). The general limits of FBA and differences between different modeling approaches might be a suitable topic for a future review article.
>
> Nonetheless, we agree that a discussion to highlight the limits of FBA, and possible extensions, would be helpful for readers (in addition to the cited reviews, e.g. [Bruggeman et al, 2023], that also discuss shortcomings of FBA).
>
> We therefore extended the description of constraints in Section 2.6, and provide a more elaborate discussion of FBA in the Materials and Methods.

Specifically, within the M&M (Section 4.2) we now write: "*Bounds on reaction rates may specify maximal uptake rates of (external) nutrients, and allow us to set minimal rates of intracellular reactions that would otherwise carry zero flux, such the rate of the RuBisCO oxygenase reaction. In our simulations the effective rate of light uptake J^*_I serves as an upper bound for the photon uptake rate, corresponding to the sum of photon utilization rates at photosystem I and II. Uptake of other nutrients is not constrained. Additional intracellular minimal fluxes are summarized in Table 1. We note that within the framework of FBA, reactions like non-growth associated maintenance and electron overflow through the terminal oxidases and the Mehler and Mehler-like reactions are set as (constant) flux bounds and do not emerge from the model. Hence the model, in its current form, cannot dissect their specific role as tolerance mechanisms under high light intensities.*"

Finally, readers would benefit from more detailed information. For instance, the experimental setup is mostly referenced and only the light intensity and composition is provided in the manuscript. Some paragraphs are vague and devoid of meaningful information, for instance, Section 2.1, paragraph 5. Did the authors perform experiments as in Zavřel et al. or just copied data from Zavřel et al.? If the former, please include a section in Material and Methods; if the latter, please briefly describe the experimental setup and recovered data in Results, especially the medium composition.

→ We agree that information on the experimental setup was (too) sparse. We therefore added an additional section in the Materials and Methods that details the relevant information, i.e., a brief overview on which data were used, how it was obtained, incl. media (BG-11), nitrogen source, etc. See new subsection 4.3 in Materials and Methods "*Experimental data used in the analysis*".

The MEMOTE report shows that three reactions are missing a GPR. Although it is highly suspicious that such a low number of reactions to have no GPR, the reactions reported are RBPC (RuBisCO Carboxylase), BM0010 (Glycogen component of biomass reaction), RBCh (RuBisCO Oxygenase). The authors associated "Biomass" as GPR in BM00XX reactions, but RBPC and RBCh are clearly enzymatic reactions.

→ This was indeed a mistake in the preparation of the file. We apologize for the mistake. For reactions without a GPR, the GPR from the previous reaction was used instead, resulting in a suspicious (and erroneous) low number of reactions with no GPR, as well as incorrect (and at times confusing) GPR. In the revised version 116 reactions have no GPR. For the reactions RuBisCO Carboxylase (RBPC) and RuBisCO Oxygenase (RBCh) the corresponding genes were added. Non enzymatic GPRs (Biomass, Spontaneous, …) were removed

Following on this, the authors reported the modeling of the Entner-Doudoroff pathway, but KEGG reports no known Synechocystis gene with EC 4.2.1.12 activity. Similarly, KEGG reports

no known Synechocystis BioC and BioH enzymes (biotin pathway), and there are unknown enzymes in the phenylalanine, tyrosine, tryptophan pathway.

> → As stated above, there was an unfortunate error in the script. BioC, BioH and other reactions were falsely assigned with genes, reassigning the last gene instead of correctly assigning no GPR. These incorrect GPR were removed.
> However, the gene slr0452 was already reported as EDD (EC 4.2.1.12) in Chen et al. (2016) and Bachhar et al. (2022). It is not currently annotated in KEGG (but is annotated in MetaCyc).

Supplementary File S5 lacks information supporting new reactions and their gene associations. For instance, slr1229 is associated with transport reactions from cytoplasm to carboxysome (and vice versa), however, KEGG/UniProt/Cyanobase agree that slr1229 can be a sulfate permease. Similarly, why is an aquaporin (slr2057) associated with PQ transport. Moreover, why are “slr1908 or slr1841 or sll1271 or sll1272 or sll0772 or slr0042 or sll1550” associated with many, unrelated transport reactions (e.g., slr1908 is annotated as hypothetical in KEGG)?

> → The comment relates to the same unfortunate error in the script. The error resulted in multiple reactions with the same GPR. The problem is now corrected, the respective reactions are now without a GPR, Slr1229 is now only sulfate transport, slr2057 only aquaporin.
> “slr1908 or slr1841 or sll1271 or sll1272 or sll0772 or slr0042 or sll1550” are outer membrane proteins/porins and transport across the outer membrane is annotated with these genes.

MEMOTE reports charge balance to be correct for 59.2% of the reactions, many involving ATP hydrolysis that miss a proton to balance the reaction charge. This clearly shows that metabolite formulas are incorrect (e.g., ATP [neutral] formula is $C_{10}H_{16}N_5O_{13}P_3$, but the nominal charge in the model is -4, so the [charged] formula in the model should be $C_{10}H_{12}N_5O_{13}P_3$).

> → Indeed, as in previous reconstruction (Knoop 2013, 2015), the (neutral) metabolite formulas were sourced from KEGG and did not correspond to the nominal charges (the latter were reported for pH=7.5). This is now resolved, all reactions (except biomass and exchange reactions) are now fully mass and charge balanced. In the process also the fatty acid synthesis was revised (removing pools that prevented correct balancing of mass and charge). FA synthesis was revised following iSynCJ816 (Joshi et al. 2017).

There is also a discrepancy as MEMOTE reports 830 reactions (not 831 as in section 2.1, second paragraph), and intriguingly, it reports only 20 mass unbalanced reactions (as the authors reports 778 are mass balanced intracellular reactions). As I can check 44 reactions are

exchange reactions, how many transport reactions are in the model? Are they mass balanced as well?

→ The original submission had 830 (the number 831 mentioned in the text, section 2.1, was incorrect). The excel file had 831 rows (but the first row is a header, which may have caused the mistake). The revised network has 920 reaction (921 rows in the excel file). The reconstruction contains a total of 70 transport reactions (all mass balanced, referring to all transport within the cell (compartments) as well as to/from the medium). There are 38 exchange reactions (i.e, reactions that connect the model, including the medium, to the “external world”). Exchange reactions are not mass balanced (as per SBML convention).
In addition, there are 7 (mass balanced) reactions in the extracellular medium. Generally, all reactions, except exchange reactions and biomass formation, are mass balanced. We note that not all compounds have a mass (e.g. photons).

Model in Supplementary File S2 has 830 reactions, while the model in S6 has 831. Please clarify.

→ The original submission had 830 (the 831 mentioned in our main text was incorrect). The excel file had 831 rows (the first row is a header, which may have caused the mistake). The revised network has 920 reactions (921 rows in the excel file).

Section 2.1, second paragraph, states stoichiometric matrix consistency was checked, but the authors did not mention the test actually failed.

→ We are not able to establish why the test fails (all reactions except biomass and exchange reactions are charge and mass balanced). The description of the testing algorithm states that all molecular masses must be positive. But there are (necessarily) metabolites without mass (either due to non-specific components, denoted as ‘R’, that are later recovered, or because no mass can be assigned, e.g. photons). We think this might be a reason the test fails (this is now also noted in the Materials and Methods, section 4.1: “*We note that, while all reactions but exchange and biomass reactions are fully mass and charge balanced, the stoichiometric consistency test fails, we assume because of compounds without an assigned mass, such as photons.*”).

Using cobrapy, the model's medium property warns that it cannot “identify an external compartment by name and choose one with the most boundary reactions”. Metabolites suffixes are not consistent with model.compartments.

→ Fixed. Cobrapy should now be able to correctly identify the external compartment by name. The naming was changed to "c": "cytosol", "e": "extracellular space", "p":

"periplasm", "u": "thylakoid", "cx": "carboxysome", "um": "thylakoid membrane", "cm": "cytosolic membrane". Abbreviations have been added in Section 2.1.

Please, provide a constrained model. Supplementary File S2 simulates a growth rate of 53.24 h-1. Moreover, please provide the medium composition in the manuscript (Details are referenced to Zavřel et a.l). Why are arginine (-114.882025 mmol/gDCW/h), histidine (-4.561751 mmol/gDCW/h), “glycogen granule”, and 2-(beta-D-Glucosyl)-sn-glycerol in the model's medium? Is the nitrogen source not nitrate as it is commonly used in the BG-11 medium? Section 2.1, paragraph 4, shows a stoichiometric balance if nitrate is used as nitrogen source, but the unconstrained model shows no uptake of nitrate, but rather intake of amino acids.

→ We have added a constrained model to the Supplemental Files (*Synechocystis_6803_constrained.xml, Suppl. File S3*). We note that SBML files are often not constrained but the constraints are set as part of the simulation. The model itself is capable of taking up a variety of nutrients, including amino acids and several N sources (some nutrients are also intracellular to e.g. allow for utilization of glycogen, if needed). During the simulation, we then specify the limits on uptake reactions for the respective simulation (e.g. nitrate as the only N source). All reported simulations use BG-11 medium.

Section 2.1, paragraph 5, shows vague details about the “obligatory byproducts” and the source of them (e.g., dialurate comes from the oxidative reduction of FMN). Please, add the necessary details for clarity and to show current gap knowledge in the Synechocystis’ metabolism that could be interesting to address.

→ We now identify the byproducts directly in Section 2.1, Supplemental Table S2 now also provides the (three) reactions they are synthesized in: Dialurate results from the oxidative reduction of FMN (Riboflavin-5-phosphate), necessary for vitamin B12 synthesis. Synthesis of 4-Amino-2-methyl-5-phosphomethylpyrimidine (which is required for synthesis of thiamine diphosphate) results in synthesis of CO and 5'-Deoxyadenosine. The latter is then degraded to 5-deoxy-d-ribose.

Section 2.6, paragraph 2, are αb and kd unitless parameters (text shows “[n.d.]”)?

→ Yes, both parameters are dimensionless numbers. We have removed the abbreviation n.d. and now write “dimensionless”. The parameter kd quantifies the proportionality between the rate of photon uptake flux and the rate of ATP utilization. The parameter αb quantifies the relative contribution of blue light. With respect to the latter, we have added a sentence in Section 2.4 to describe equation (7): “*... where the dimensionless parameter \alpha_b quantifies the contribution of the additional (and constant) blue background intensity relative to the intensity I_0 of red light.*”

Section 2.6, paragraph 3, the NGAM estimation error opens the possibility to obtain a negative value (i.e. production of ATP). Was the parametrization constrained to avoid this situation?

→ The parameterization was given an initial condition and an interval. We have added a sentence in the Materials and Methods (Section 4.6 Parameter estimation). The results are robust with respect to the choice of the interval.

Section 2.6, paragraph 5, how a theoretical doubling time of 3.0 h in the absence of photoinhibition and other detrimental effects is plausible if “photoinhibition and other detrimental effects cannot be fully alleviated”?

→ The value of 3.0 h is an extrapolation from the fitted parameters. In the model, the parameter KL has the role of a “maximal capacity”. With this maximal capacity we can extrapolate a theoretical maximal growth rate (or minimal division time). This extrapolated value does not take photoinhibition and other detrimental effects into account (see also Figure 3, the middle panel basically corresponds to the extrapolated growth rate).

We find it interesting that the fitting allows us to infer such a “capacity” from data. The numerical value of this capacity is such that the (theoretical) minimal division time is slightly below experimentally observed minimal division times (which is plausible since no experiment can implement the full capacity without any detrimental effects). It would be interesting to estimate this capacity value for other strains or under different conditions, e.g. for fast growing strains. We are not aware of currently available data that would allow us to do so (and it would be beyond the scope of the current manuscript).

To make our reasoning clearer, we have re-written the paragraph as follows: “*Together with the (maximal) biomass yield Y \approx 1.84 to 1.97 gCDM/mol (photons) the maximal capacity K_L allows us to infer an (extrapolated) maximal growth rate of \mu^{\max} = 0.23 \, \mathrm{h}^{-1}, corresponding to a minimal division time of approximately 3.0h. However, photoinhibition and other detrimental effects prevent that the extrapolated maximal growth rate can be realized experimentally. The extrapolated theoretical maximal growth rate is slightly lower than the minimal division time reported for \textit{Synechocystis} sp. PCC 6803 observed under optimal conditions~\cite{vanAlphen2018}.*”

In addition, we now discuss the possibility to compare these values across conditions in Section 2.12 (“Application scenarios”, newly added): “*The analysis then allows us to infer additional growth properties that are not readily accessible by conventional FBA, such as the (decreasing) biomass yield as a function of the light intensity, or the maximal photosynthetic capacity K_L. It would be of particular interest to investigate to what extent the inferred parameters depend on the specific experimental conditions, and how, for example, the maximal*

photosynthetic capacity K_L (and hence the extrapolated minimal division time) differs between experimental conditions and between different strains, including fast-growing cyanobacteria~\cite{Yu2015,Jaiswal2018,Nixon2020}".

Section 2.7 lacks a corresponding Materials and Methods subsection. Also, there is a lack of details regarding how exactly dissolved oxygen was measured (only a reference to Zavřel et al. papers).

→ We have added a section to the Materials and Methods (Section 4.3) that provides a brief overview of all measurements and data used. With respect to Section 2.7, we have added a paragraph that describes how to estimate the photon flux: "*The required parameters, the incident light intensity I_0, the transmitted light intensity I_Z, as well as the areal biomass density \rho_A has been reported in Zavrel et al. [1], see Materials and Methods Section 4.3, hence we can calculate J_I^{PBR} directly from data*".

We currently do not provide the derivation of equation (8) as this has been detailed in the cited literature (Huismann et al., 2002, and Steuer 2023), but can add a respective section in the Materials and Methods at the request of the reviewer or editor.

While the work is of great importance and interest to a broad audience, the quality and presentation of this works needs to be improved prior to publication.

→ We thank the reviewer for the positive evaluation, and apologize for the mistake in the annotation of the network. We hope the work has improved significantly during the revision.

MINOR COMMENTS:

Is there a reason to use "GSMR" and not GSMM or GEM, as the latter are commonly used as acronyms for genome-scale metabolic models?

→ We commonly use GSMR for genome-scale metabolic reconstruction, whereas GEM and GSMM stands for genome-scale metabolic model, to distinguish between a "reconstruction", and a "model". The latter may include hypothetical reactions (to ensure functionality) or auxiliary reactions. The abbreviation GSMR only occurs when referring to the reconstruction (within the Introduction). We are happy to switch notation if PLoS has a particular preference.

Is there a reason to provide a reduced model? The reduced model depends on the protected reactions that could be different for another application of the scope of the provided reduced model.

→ The reduced model was meant as a supplementary addition for readers. We agree that it plays no role in the current work, and we have therefore decided to remove it from the present manuscript. We also agree that the protected reactions do indeed depend on the applications, and hence should be motivated and discussed properly when provided.

The authors included the MEMOTE report for the whole developing branch. I locally run MEMOTE and, although the results seem to agree with Supplementary Text S6, model data should be corrected. For instance, the authors report the use of SBOannotator, however MEMOTE reports that only 14.3% of the reactions contains “SBO:0000176”. Annotation can be added manually.

→ As noted above, there were some unfortunate errors in the submitted files. The issue above, however, is a known problem in MEMOTE also reported by others (as of spring 2024): SBO annotation follows a hierarchic style. The SBO:0000176 (biochemical reaction) entry has several descendents (e.g. SBO:0000376 hydrolysis). The MEMOTE testing suite only tests for presence of the “parent” SBO:0000176, although the use of descendents is more accurate.
This is a known issue: https://github.com/opencobra/memote/issues/469 that also others faced, e.g. [Feierabend et al. (2021). High-quality genome-scale reconstruction of Corynebacterium glutamicum ATCC 13032. Frontiers in Microbiology, 12, 750206]: “The current version of MEMOTE checks every reaction for the annotation with the most general SBO term (SBO:0000176), “biochemical reaction.” This check implies that MEMOTE can not yet capture the fine-grained description of biochemical reactions in this model. Thus, the score of the metabolic reactions of 33.4 % diminishes the overall MEMOTE SBO term annotation score”

Check the enumeration of the supplementary material. The MEMOTE report appears as “Supplementary Text S6” (section 4.1), but added as “S7_TEXT-MemoteReport.html” in the submission files.

→ All files were checked for correct description and numbers.

Supplementary File S5 separator character (“|”) is not a common alternative when opening a csv file using spreadsheet software. Please, change the separator character to a tabulator or provide an Excel file rather than a text file.

→ We have changed the separator to “tab”.

Please, enumerate the text. Enumeration allows comprehensive comments regarding specific text in the document. I believe, as the document was prepared using LaTeX, the commands are “\usepackage{lineno}”, then “\linenumbers”

→ We have added line numbers in the revised text.

Supplementary Material:

Table S2 shows negative excretion flux rates, so they represent intake flux rates. Please correct the flux signs.

→ We have changed the flux signs.

Why are the model provided in Supplementary Material S2 and the model provided from Supplementary Material S6 different?

→ The models have been revised (see above). Both models should be identical.

Reviewer #2:

In their article "A quantitative description of light-limited cyanobacterial growth using flux balance analysis", the authors describe a development of a genome-scale model of the cyanobacterium Synechosystis to realistically include light harvesting, usage, and light-induced photoinhibition. The model improvement is elegant, because it allows defining additional constraints into the modelling framework of constraint-based modelling, which realistically reflect constraints of the photosynthetic machinery, and still allow treating the computational problem with linear programming. The two main constraints are i) a variable quantum yield, reflecting that at high light absorbed light energy is quenched and released as heat and ii) and light-dependent ATP requirement term, which reflects additional demand of resources in photoinhibition due to the required protein repair.

Overall, the paper is very clear and well written. However, it requires a very careful spelling and grammar check, because it is full with totally avoidable little typos, starting in the very first sentence "The metabolism of phototrophic cyanobacterial ...".

→ We thank the reviewer for the positive evaluation. We have tried to remove all remaining spelling mistakes. The first sentence (in the abstract) was indeed wrong (a last minute change from “Phototrophic cyanobacterial metabolism” to “The metabolism of phototrophic cyanobacteria …”).

Unfortunately, I was unable to execute the Jupyter notebook. This should urgently be improved before publication. See point 9 below.

→ We have revised the Supplementary materials. The Jupyter notebook might have been "overambitious", as it was designed to directly download the required data (excel file) from the eLife website. We think this may have caused problems ("ValueError: Excel file format cannot be determined").

We now instead provide two Jupyter notebooks: A simplified notebook that simulates the model and generates the respective figures for a given set of parameters (same parameters as used in the manuscript). In addition, we provide an additional separate Jupyter notebook that carries out the parameter estimation, with the data provided in a CSV file. We note that the 2nd notebook takes more time to execute, and we recommend using the first to explore the model. We hope this resolves the problem.

The typos and the issues with the notebook aside, this is a very good paper which, in my opinion, requires only minor changes. My suggestions are as follows:

1) I would like to commend the authors for determining the biomass composition systematically for different light intensities / growth rates. This is rarely found and a valuable and highly interesting experimental data set. I wonder if the authors could also give the elemental composition of the biomass. After all, for thermodynamic considerations, e.g. to find the energy of formation of biomass, only the elemental composition is required. (page 6)

→ We fully agree that an unknown or assumed biomass (often also just adopted from *E. coli*) is a major issue in many applications of FBA. We must note, however, that the measurements were already originally reported in Zavrel et al (2019), and not all biomass components were measured.
Given a biomass composition, the model indeed allows us to estimate the elemental composition of the cell, either directly using the BOF, or indirectly by considering the net exchange fluxes for growth (both give identical results).

The elemental composition is directly accessible from the stoichiometric macro equation provided in Section 2.2 ("Network properties and overall stoichiometry", the section title was added newly to split section 2.1 into smaller more accessible parts).

We now also provide the elemental composition in the Supplementary Information (Supplementary Table S3). We now have also (slightly) extended the discussion on elemental ratios in the text.

2) Not being a cyanobacteria expert, I am quite surprised by the rather high PQ ratio. If 1.4 O2 are released per CO2 absorbed, where do the additional 40% come from? The overall

stoichioimetry for carbon fixation should yield a ratio 1:1. Is the extra oxygen explainable by the reduction of inorganic nutrients (here, nitrage, phosphate, and sulfate) alone? (page 6)

→ The stoichiometry of (only) carbon fixation is indeed 1:1. The ratio of 1.4 O_2 per CO_2 assimilated comes from further requirement of reductant (mostly NADPH) for anabolism (we note that O_2 is also used as a substrate in metabolism, but the overall flux is low compared to photosynthesis). The PQ ratio in the model is in good agreement with reported values from literature, for example Pirt ("*The thermodynamic efficiency and dynamics of photosynthetic growth*", New Phytol, 1986, now also referenced) provides an overall stoichiometry of algal biomass with nitrate as nitrogen source.

$$0.71\ CO_2 + 0.59\ H_2O + 0.101\ HNO_3 = 0.71\ \text{C-mol biomass} + O_2,$$

This overall stoichiometry gives a photosynthetic quotient PQ of approx 1.41 (here we only consider nitrate as a source of N, the N source has a significant impact on the ratio due to the high mass fraction of N compared to other elements). We now also cite E. A. Laws, "*Photosynthetic quotients, new production and net community production in the open ocean*", 1991, who provides similar numbers.

3) Photorespiration: the authors set the flux of the RuBisCO oxygenase reaction to 3% of the total of the RuBisCO flux, but do not give a motivation. Where does this percentage come from? Even considering carbon concentrating mechanisms in cyanobacteria, this seems rather low. (page 10)

→ The value of 3% flux through the RuBisCO oxygenase was taken from previous publications (Knoop 2010, 2013). Since photorespiration was a major theme in previous publications, we did not repeat the detailed discussion in the present work (in brief, a residual photorespiration (in the range 3-5%) can even be non-detrimental because it can provide serine for biosynthesis (from glyoxylate). In the earlier reconstructions, photorespiration was even essential because the model lacked alternative pathways to synthesize serine. Such a pathway was later identified (also motivated by the model, see Klemke et al. (2015) "Identification of the light-independent phosphoserine pathway as an additional source of serine in the cyanobacterium *Synechocystis* sp. PCC 6803", PMID: 25701735). A ratio of 3-5% is consistent with experimental estimates (some works also report much lower values, e.g. Jamey D. Young et al. (2011), "*Mapping photoautotrophic metabolism with isotopically nonstationary 13C flux analysis*" Metab Eng. 13(6), who report negligible photorespiration (<0.5%), albeit under high CO_2 conditions).
We have added the respective references to the section and now write:
"*Following previous models [5], the flux is set to 3% of the total RuBisCO flux, within the range of experimental estimates [44, 45]. See also Knoop et al. [5] for a discussion of photorespiration in the context of FBA.*"

4) The experimental conditions are very specific, and while it can be expected that fitted parameters differ under different light conditions, possibly better reflecting natural conditions, the basic findings are very likely of general validity. Nevertheless, it would be nice to have a more elaborate discussion on how the model would have to be expanded in order to realistically simulate different light spectra. (page 12)

> → We fully agree that the based findings are likely of general validity. For example, as pointed out in the response to Reviewer 1, the model allows us to estimate a maximal capacity of the strain and it would be interesting to estimate this capacity for other strains or under different conditions, e.g. fast growing strains.
> To account for possible applications and extensions to different light spectra, we have added an additional section that outlines possible application scenarios, including an extension to different spectra (Section 2.12 “Application scenarios and further development”) in which we discuss the possible extensions of the model (albeit briefly, as it is beyond the scope of the current model). In addition, we have re-written parts of the “Conclusions” to highlight the key results from our analysis.

5) The shaded area in Fig. 5 "[...] reflects the weight of blue photons from 0 to 100%." This is not clear to me. Earlier it was argued that a relative efficiency of 63% fitted the data best, so why is this necessary here? Moreover, why are there two shaded areas (light and dark blue)?

> → Indeed, the value 63% fitted the data best (and is used for the fitted model, also in Fig 5). However, the shaded area refers to the “light uptake” estimated using the geometry of the reactor vessel and the Lambert-Beer (LB) equation. This estimation is entirely independent of the fitting processes, and Figure 4 serves to compare both estimates. Hence we did not want to bias the comparison by using the fitted value also for the LB equation (which is unrelated to any fitted parameter).
> The two shaded areas indicate uncertainty from the contribution from blue light (0-100%), as well as the uncertainty due to the error in estimating the biomass density in the reactor (measuring dry mass is often associated with significant error, and the LB equation requires knowledge of the dry weight per volume or area). We have also revised the figure to enhance readability. Colors are now consistent with other Figures: Black for experiments (here dark and light shaded areas) blue for simulation.

6) "we note that photodamage is modelled as ATP utilization only, and therefore might overestimate the ATP, and hence photon requirements, at high light intensities" - this is very plausible. Even with a photoinhibition rate proportional to light intensity, the additional repair rate (and hence the additional ATP consumption) would not increase linearly, because in higher light the proportion of already damaged reaction centers increases, so that a smaller fraction can receive damage. This was, for example, shown in a recent mechanistic, differential

equations-based model on photoinhibition (https://doi.org/10.1101/2023.09.12.557336). (page 14)

→ We agree. We also emphasize that we do not claim that our approach represents a detailed mechanistic model of photodamage. Rather it is a mechanistically-motivated extension of FBA to better capture properties of phototrophic growth, without sacrificing the simplicity of FBA. We also extended the discussion with respect to detailed mechanistic models of photosynthesis and the possibility of hybrid models (see below). Due to comments of another reviewer, we have also reduced the depicted range of light intensities in Fig 7, such that there is less emphasis on high light intensities.

7) Figure 7 shows the simulated ATP/NADPH ratio and some experimental data points. It would also be interested, which ratio of CEF to LEF the model predicts, because also these can, in principle, be measured separately.

→ Supplemental Figure S3 provides the predicted CEF to LEF ratio. We decided to use the ATP/NADPH ratio in the main text to facilitate a direct comparison with the discussion of Theune et al. (2021).

8) In the conclusions, the authors claim that their "[...] method is motivated by mechanistic models of photosynthesis". However, as far as I understood, this pertains only to a mechanistic model of light absorption (section 2.7). However, there is a huge amount of literature on detailed mechanistic models of photosynthesis, and it would be interesting to see how the model results presented here compare to these. That said, I do not expect the authors to perform numerous dynamic simulations with published models, but this body of literature should also not be completely ignored. Several assumptions and observations can very well be justified by also looking into mechanistic dynamic models. A very comprehensive review on this subject was published by Stirbet et al, 2020 (https://doi.org/10.1093/aob/mcz171).

→ We agree. As noted above, we do not claim that our approach represents a detailed mechanistic model of photosynthesis. Rather, our aim is to provide a "better FBA" using a minimal amount of additional parameters (we note that all mechanistic models require extensive parameterization and almost all parameters, even when available from the literature, also depend on detailed experimental conditions and may change, e.g., as a function of temperature. Instead we rely on empirical parameters that can be estimated from a given growth-irradiance curve. To discuss this aspect, we have added a section 2.12 "Application scenarios and further developments").
In addition, we have re-written parts of the Conclusions, to also discuss the possibility of hybrid models (in Conclusions):
"*Hence, we consider our approach to be an intermediate method between FBA and more complex models of light absorption and phototrophic growth, e.g., genome-scale ME/RBA models [...] or hybrid models that combine detailed kinetic descriptions of photosynthesis with*

stoichiometric models of metabolism [Westermark2016]. In particular, while hybrid models than incorporate a detailed mechanistic differential equations-based models of photosynthesis are a promising path towards an improved description of phototrophic growth, such models also require a significant additional investment with respect to the required knowledge of parameters, and give rise to a significantly increased computational complexity."
We also cite Westermark (2016) and a general discussion on different modeling approaches (while already from 2016, most of the issues in there remain valid today), and do not want to go too deep into kinetic models of photosynthesis. We see our approach primarily as an "improved FBA", rather than a replacement of more complex models.

9) The Jupyter Notebook unfortunately failed in the third cell, during the import of modules.zavrel_FIT with the error message "ValueError: Excel file format cannot be determined, you must specify an engine manually." Documentation of the code is insufficient to solve this problem. Please improve the notebooks and the documentation as necessary.

→ See above. We hope the issue is now fixed.

Reviewer #3:

The authors of this paper aim to enhance our understanding of cyanobacterial metabolism and growth, which play a crucial role in global biogeochemical cycles and have potential applications in sustainable biotechnology. They present an updated reconstruction of the metabolic network of the cyanobacterium Synechocystis sp. PCC 6803 and analyze it using flux balance analysis (FBA).

To overcome the limitations of conventional FBA and integrate quantitative experimental analyses, they propose a novel method for describing light absorption and utilization. This method incorporates photoinhibition and a variable quantum yield into the constraint-based description of light-limited phototrophic growth.

The authors demonstrate that their model can predict quantitative properties of cyanobacterial growth, including photosynthetic oxygen evolution and the ATP/NADPH ratio required for growth and cellular maintenance.

The manuscript would be suitable for publication in PLOS Computational Biology after addressing the comments provided below:

→ We thank the reviewer for the positive evaluation.

Figure 2 is inserted in the text without context. The manuscript would benefit from a better connection between the text and the different parts of this figure.

→ The figure summarizes the experimental data used in the analysis. We agree that the description was too brief, and have now added an additional section in the Material and Methods that explains the data used in the analysis. In addition, we have modified the text to better embed Figure 2 (also with a reference to the new section in the Materials and Methods).
We now write: "All analysis is based on a coherent set of growth experiments, reported previously [1] and summarized in the Materials and Methods, section~\ref{sec:data}. Measured quantities include the specific growth rate as a function of light intensity, the respective changes in biomass composition, light-dependent oxygen (O$_2$) evolution, as well as O$_2$ consumption in darkness. Figure 2 summarizes the data".

The authors claim that their model is usable even when information regarding the light spectrum and geometry of the reactor vessel is lacking. However, only data from one experimental setup was used to validate their simulation results. The authors should better discuss the potential limitations of their model considering the specific conditions used to validate the model.

→ We agree. As also pointed out by reviewer 2, while it can be expected that fitted parameters differ under different light conditions, the method and its basic findings are of general validity. For example, the model allows us to estimate a maximal capacity of the strain and it would be interesting to estimate this capacity for other strains or under different conditions, e.g. fast growing strains.

To account for possible applications of our approach in different settings, we have re-written parts of the Conclusions and added an additional section (Section 2.12 "Application scenarios and further development") that discusses how to use our approach in different scenarios (in particular when information about the experimental setup is lacking and only a growth-irradiance curve is available).

The Conclusions now also distinguish between the present analysis (with detailed experimental data available) and applications where such data might not be available:
"*The key conclusions of our work are twofold. Firstly, we have shown that the metabolic reconstruction, together with the available quantitative data, provides a consistent description of phototrophic growth under the given experimental conditions. That is, light absorption, O$_2$ evolution in light, O$_2$ consumption in darkness, biomass yield, ATP/NADPH production, and growth rate together provide a quantitative and consistent overall picture of cyanobacterial energetics, [...]*
Secondly, our approach extends conventional FBA, while retaining most advantages of FBA in terms of computational simplicity. Our approach is applicable in all situations where a (usually experimentally measured) growth-irradiance curve is available that allows for the estimation of the three parameters α, K_L, and k_d (see Section 2.12 "Application

scenarios and further developments"). Our approach then provides additional information, such as [...]."

Some assumptions and discrepancies between experimental results and simulation values should be better explained. For instance, in Figure 7, the experimental values for the ATP/NADH ratio are only available for light intensity lower than approximately 300. How can the authors claim that the predicted and experimental values are in good agreement without addressing the dataset limitations?

→ We fully agree that the claim of "good agreement" only holds for the measured range (which covers medium light intensities up to 300muE/m^2/s) and not for higher light intensities.

We have therefore modified the text and now write: "*Nonetheless, within the range of experimentally used light intensities (up to $I_0 \approx 300 \, \mu E/m^2/s$), the predicted and experimental values are in good agreement, indicating …*".

Furthermore, we have reduced the range of the x-axis to 600 \muE/m^2/s to show a smaller range. Still, we consider the higher light intensities (at least up to 600) also of relevance, as the model suggests an increase in the ATP/NADPH ratio that could be tested experimentally.

We now write: "*As a potential test for further analysis, the model suggests that the ATP/NADPH ratio will increase with increasing light intensities, primarily due to the increased requirement of repair mechanisms to alleviate photodamage*".

Did the authors consider a hybrid model to better simulate the dynamic behavior of photosynthesis while maintaining a relatively less complexity than kinetic models?

→ Such a simulation would be interesting, but is beyond the scope of the present work. We also emphasize that even rather small dynamic models of photosynthesis already give rise to a large number of kinetic parameters (that are often not constant but are also highly condition dependent and may change, e.g., with temperature). In contrast, our analysis has only 4 main parameters (alpha, ab, KL, kd) and 2 additional parameters (NGAM, vOXmin) that fully parameterize the model (in most applications of FBA, the latter 2 are given as external constraints). The purpose of our work is to show that such a parameterization can already provide a reasonable description of key physiological parameters (e.g. growth rate, yield, …), and improves upon the use of FBA for phototrophic microorganisms, while still keeping the computational simplicity and wide applicability of FBA.

To better emphasize our reasoning, we have extended the discussion and now write (in Section Conclusions):

"*Hence, we consider our approach to be an intermediate method between FBA and more complex models of light absorption and phototrophic growth, e.g., genome-scale ME/RBA models [] or hybrid models that combine detailed kinetic descriptions of photosynthesis with stoichiometric models of metabolism [Westermark2016]. In particular, while hybrid models than incorporate a detailed mechanistic differential equations-based models of photosynthesis are a promising path towards an improved description of phototrophic growth, such models also require a significant additional investment with respect to the required knowledge of parameters, and give rise to a significantly increased computational complexity.*"

We also cite Westermark (2016) and a general discussion on different modeling approaches (while already from 2016, most of the issues remain valid today), and do not want to go too deep into kinetic models of photosynthesis. We see our approach primarily as an "improved FBA", rather than a replacement of more complex models.

Minor comments:

Some italics are missing in the names of species.

→ We re-checked for consistent notation. Species name is now always in italics.

The plot in Figure 5 contains different blue lines. The image would benefit from different colors for different conditions; otherwise, it is confusing to follow the description.

→ The figure has been revised.

The same goes for the plot in Figure 6, which utilizes the same color for model and experimental results. Different colors would make the plot easier to interpret.

→ The figure has been revised.

Reviewer #4:

In this manuscript, the authors propose a computational framework tailored specifically for characterizing the growth dynamics of phototrophic microorganisms. By incorporating mechanisms of light absorption, utilization and photoinhibition, the approach offers a more comprehensive understanding of how metabolic processes respond to varying light conditions. The authors claim that this novel computational framework represents a significant advancement in the quantitative analysis of light-limited phototrophic growth. Furthermore, the

authors argue that its applicability extends beyond cyanobacteria, offering insights into the growth dynamics of diverse phototrophic microorganisms.

I have no major comments as this manuscript is well written and the approach is new, to the best of my knowledge, and could be used in a lot of different modeling works.

→ We thank the reviewer for the positive evaluation.

I have a few minor comments, especially about the figures, that could help improve the comprehensibility of the manuscript a little bit.

The authors describe the creation of a reduced core model and provide this model, but they never really use it in their study. At the end of the introduction, I was really expecting to see it in the manuscript and was quite disappointed not to see it. Is it really interesting to include it in this manuscript?

→ The reduced model was meant as a supplementary addition for future analysis. But we agree that it plays no role in the current work, and we have therefore decided to remove it from the present manuscript.

Regarding the definition of the light-dependent BOF, the authors mention some "by-products", three of them containing carbon. It would be nice to have the list of them, even though a reader could find them in supplementary S2. Could those byproducts be linked with inconsistencies in the topology of the network?

→ We now list all byproducts already in the main text, and only refer to the Supplementary Table S2 for further details. Within the network, the byproducts are generated in two essential reactions (plus a reaction that degrades one intermediate). Deletion of the respective reactions prevents growth. We do not know if there are further (currently unknown) reactions that may recycle the byproducts back into metabolism. We also have extended the Supplementary Table S2 to include the respective reactions in which they are synthesized.

(We note that it is an interesting scientific question to what extent recycling of byproducts by expression of additional enzymes is actually a positive net carbon/energy gain for growing cells, as the expression of the necessary enzymes also requires resources. But this question is beyond the scope of the current work).

The description of the equation is well done with the different parameters well explained, except for the Haldane/Aiba equation for which we know what "I" is, and that γ is dimensionless, but that's it. A little more explanation would be great.

→ We have revised the text. The parameters in the Haldane/Aiba equation are primarily phenomenological (i.e. fitted parameters). In the publication of Aiba (1982, among the first to suggest this equation to describe phototrophic growth) they are called “empirical parameters” and are labeled a, b, c (later also semi-mechanistic derivations of the equation were proposed).

To explain the equation and its parameters, we now point out that, in the absence of photoinhibition (gamma = 0), the Haldane/Aiba equation is identical to the Monod equation, with the parameters KA and \mu^* (the half-saturation constant and the maximal growth rate). In the presence of photoinhibition, the values do not exactly correspond to the half-saturation and maximal rate but they describe similar properties. We think the equation is best understood in comparison with the (well known) Monod equation.

Therefore we now write: ”*The Haldane/Aiba equation is specified by three empirical parameters, \mu^*, K_A, and \gamma. The parameter γ is a dimensionless number that quantifies the inhibition of growth with increasing light intensity (photoinhibition). In the absence of photoinhibition (\gamma = 0), the equation is identical to a Monod equation with a half-saturation constant K_A and a maximal growth rate \mu^*.*”

I think the figures could benefit from further refinement to improve their clarity and comprehensibility:

- In Fig. 1, the blocks of color on the right representing the anabolic reactions are quite disturbing, since the authors use the same colors as the ones in the figure. I started wondering why the thylakoid membrane was referred to as "Cell wall" in the legend.

→ We have revised the figure (indeed, the colors of the biomass components were unrelated to the remaining figure).

- In Fig. 2, the width of the error bars is way too small in panels A, D, and E. I reviewed the manuscript after printing it, and they were barely visible. Even on the PDF, the error bars are way too small. The exact same goes for Fig. 3.

→ We have increased the width of the error bars (and revised figures throughout)..

- In Fig. 4, while I understand that the blue line is wider at the beginning to show the contribution of blue photons, I am pretty sure that there would be a better way to show that.

→ We have revised the figure. However, we are not aware of an obvious better way to show the contributions of the blue photons. We would like to keep the uncertainty relating to the blue photons within the plot (the effect is small).

- In Fig. 5, I would have chosen different colors to display the three "lines". It is especially an issue regarding model VS experiment.

→ We have revised the figure to use different colors. Colors are now consistent with other Figures: Black for experiments (here dark and light shaded areas) blue for simulation.

Regarding the supplementary material, I appreciate the fact that all data and python files are available, and that the metabolic networks are full of cross-references. I noticed that the SBML model provided in supplementary material S2 and the one provided in supplementary material S6 are not the same, **with one reaction of S6 (R_PP0011) apparently split into two different reactions in S2 (R_RBPC and R_RBCh), and R_BM0010 being absent from S6.** Could the authors explain why and the possible implications on the results?

→ All supplemental files were revised. The files should be consistent now. The RuBisCO carboxylation and oxygenation (two functions of the same enzyme) can indeed be either split into separate reactions or as a single overall reaction with a fixed ratio. We now opt for the former (with a flux constraint that enforces flux through the oxygenase).
