# Supplementary material for: A quantitative description of light-limited cyanobacterial growth using flux balance analysis
Source: PLoS Comput Biol. 2024 Aug 5;20(8):e1012280. doi: 10.1371/journal.pcbi.1012280 (PMC11326710; doi:10.1371/journal.pcbi.1012280)
Supplement: Attachment — Submitted filename: Response-Reviewers - PCOMPBIOL-D-24-00071R1-TEXT.pdf [file pcbi.1012280.s009.pdf]

**Response to the reports of the reviewers on the revised manuscript "A quantitative description of light-limited cyanobacterial growth using flux balance analysis" submitted to PLoS Computational Biology (PCOMPBIOL-D-24-00071R1)**

We would like to thank the reviewers for their careful evaluation of our revised manuscript.

Below, we provide a response to all comments and describe the respective changes in the manuscript. The original reviews are included unabridged, our response is indented and highlighted in blue. All changes are also marked in the original manuscript.

## Comments to the Authors:

**Reviewer #1:**

The authors have now addressed all previously raised points. Please find below comments for this revised version to consider.

MAJOR COMMENTS:

MEMOTE does not use formulas to determine if the (stoichiometric) matrix is consistent (see https://github.com/opencobra/memote/blob/develop/src/memote/support/consistency.py, lines 85-108), but I agree that photons cannot be (mass) balanced in the model and that leads to the result. In fact, Supplemental File S6, field "Minimal Inconsistent Net Stoichiometries" shows the inconsistency arises from photons imbalance, but also protons.

> → The inconsistencies due to protons have been resolved. The remaining "inconsistencies" as reported by MEMOTE are due to the mass-less photons.

MINOR COMMENTS:

Please consider another format for references in Table 1. It looks like the reference "2" is the exponent ("hours square"). Maybe add another column with the references.

> → We have added another column in Table 1 and changed the format.

There are small typos, like "and and ranges from 1.84 to 1.97 gCDM/mol photons for the light-dependent BOF" in page 6, "was shown to less efficient" in page 11, "prepheneate" in page 22.

> → corrected.

Although it is detailed that all the experimental data comes from Zavřel et al, I think that "Specifically, in addition to the O2 evolution as a function of the light intensity, the O2

consumption shortly after onset of darkness was measured” on page 9 still need to be referenced.

→ We added the reference after the sentence.

I think it is a stylistic choice, but I would prefer “free parameter” rather than “adjustable parameter” (as I noted a change in page 10: “with KL as an additional free parameter”). Other choices such as “GSRM” instead of “GSMM” can be settled by the editor.

→ We agree and changed all occurrence of “adjustable parameters” to “free parameters”.

In page 10, values should match the order of the parameters in the text: “The estimated parameters for the terminal oxidase and the NGAM reaction are vmin OX = 9.1 ± 9.04 [%O2] and vmin NGAM = 1.45 ± 0.48 [mmol/gCDM/h], respectively”

→ We have changed the order.

Another choice is “Synechocystis 6803” rather than “Synechocystis sp. PCC 6803”. The former is only used two times (page 10 and 22), while the second should be used throughout the manuscript.

→ We changed all occurrences to Synechocystis sp. PCC 6803.

On page 22, “N” is not defined. Usually, “S” is employed to denote the Stoichiometric matrix.

→ We now also define N (“FBA is based on knowledge of the stoichiometry of metabolism, as given by a stoichiometric matrix N, … ”). We are aware that both N and S are used to denote the stoichiometric matrix. Here, we use N, following the works of Stefan Schuster, Reinhart Heinrich and others.

It might be helpful to add markers to Figure 4, showing exactly the tested light intensities. This was done for Figure S4 and others. Similar comment for Figure 5.

→ We have changed the figures accordingly and now indicate measured light intensities with a bar.

Is it not better to combine Figure 6 and Figure S2 to improve the comparison of model simulations? Also, the description of Figure 6 reads “an offset of ≈ 12%” while Figure S2 says “deviation of 12%” (missing the “≈” sign)

→ We now have combined Figure 6 and S2 into a single figure (as Fig 6 in the main text). The description was changed to an “an offset of 12%” (the experimental expectation is approximately 12%, but the line is, of course, exactly 12%).

Is there a missing value in Figure 6 and Figure S2? Or oxygen exchange and dark respiration were not measured at 1100 μE/m2/s? Would these missing values affect the parameterization?

→ Indeed, the oxygen exchange was not measured at all values for which growth was measured. However, the missing values have no (significant) effect on the parameterization (the precise numerical values will be slightly different, of course). In particular, the missing values follow the expectations given by measured values. This fact was also ascertained by comparing the values to earlier measurements (reproducibility of the setup was one of the major themes in reference [1] from which the data was obtained. The figure below is sourced from Figure 1 from reference [1]). For the current analysis, we preferred to stay within a single dataset.

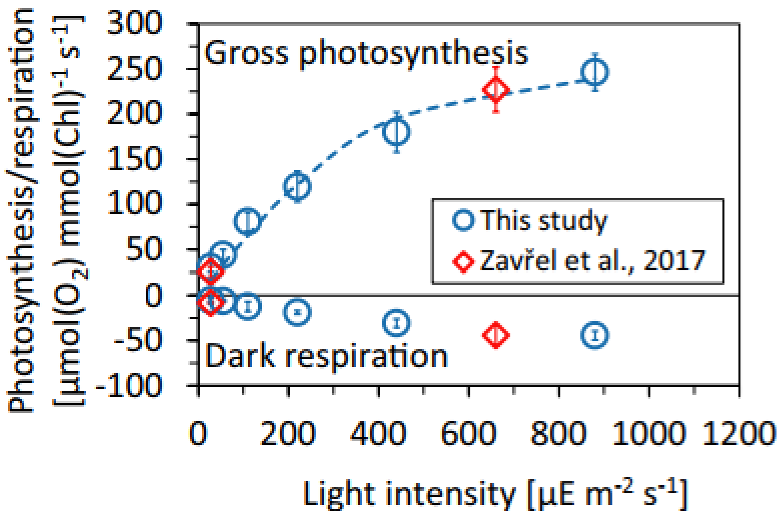


**Reviewer #2:**

The authors addressed all comments to the reviewers to my full satisfaction.
